# ESTES guidelines: acute mesenteric ischaemia

**DOI:** 10.1007/s00068-016-0634-0

**Published:** 2016-01-28

**Authors:** J. V. T. Tilsed, A. Casamassima, H. Kurihara, D. Mariani, I. Martinez, J. Pereira, L. Ponchietti, A. Shamiyeh, F. al-Ayoubi, L. A. B. Barco, M. Ceolin, A. J. G. D’Almeida, S. Hilario, A. L. Olavarria, M. M. Ozmen, L. F. Pinheiro, M. Poeze, G. Triantos, F. T. Fuentes, S. U. Sierra, K. Soreide, H. Yanar

**Affiliations:** Surgery Health Care Group, Hull and East Yorkshire Hospitals NHS Trust, Hull, UK; Emergency Department, Istituto Clinico Città Studi, Milan, Italy; Emergency Surgery and Trauma Unit, Humanitas Research Hospital, Rozzano, Italy; Department of General Surgery, Ospedale di Legnano, Milan, Italy; Servicio de Cirugía General y Digestiva, Hospital Universitario de Torrevieja, Torrevieja, Spain; Surgery 1–Tondela-Viseu Hospital Centre, Viseu, Portugal; Department of Surgery, Milton Keynes Hospital NHS Foundation Trust, Milton Keynes, UK; 2nd Surgical Department, Kepler University Clinic Linz, Linz, Austria; Division of Trauma and Acute Care Surgery, Mafraq Hospital, Abu Dhabi, United Arab Emirates; Department of Angiology and Vacular Surgery, University Hospital of Torrevieja, Torrevieja, Spain; Department of General Surgery, Centro Hospitalar de Vila Nova de Gaia/Espinho, Vila Nova de Gaia, Portugal; 2nd Surgical Department, Santo André Hospital, Leiria, Portugal; Servicio de Cirugía General y Digestiva, Hospital Galdakao Usansolo, Vizcaya, Spain; Department of Surgery, Medical School, Hacettepe University, 06100 Ankara, Turkey; General Surgery Department, Hospital São Teotónio, Viseu, Portugal; Department of Surgery/Intensive Care Medicine, Maastricht University Medical Center, Maastricht, The Netherlands; Department of General Surgery, Rhodes General Hospital, Rhodes, Greece; General Surgery 2 and Emergency Surgery, University General Hospital Gregorio Marañón, Madrid, Spain; Department of Surgery, Galdakao-Usansolo Hospital, Galdakao, Vizcaya Spain; Department of Clinical Medicine, University of Bergen, Bergen, Norway; Department of Gastrointestinal Surgery, Stavanger University Hospital, Stavanger, Norway; Department of General Surgery, Istanbul Faculty of Medicine, Istanbul University, Çapa, Istanbul, Turkey

**Keywords:** Acute mesenteric ischaemia, Diagnosis, Clinical management, Guidelines

## Abstract

**Purpose:**

Acute mesenteric ischaemia (AMI) accounts for about 1:1000 acute hospital admissions. Untreated, AMI will cause mesenteric infarction, intestinal necrosis, an overwhelming inflammatory response and death. Early intervention can halt and reverse this process leading to a full recovery, but the diagnosis of AMI is difficult and failure to recognize AMI before intestinal necrosis has developed is responsible for the high mortality of the disease. Early diagnosis and prompt treatment are the goals of modern therapy, but there are no randomized controlled trials to guide treatment and the published literature contains a high ratio of reviews to original data. Much of that data comes from case reports and often small, retrospective series with no clearly defined treatment criteria.

**Methods:**

A study group of the European Society for Trauma and Emergency Surgery (ESTES) was formed in 2013 with the aim of developing guidelines for the management of AMI. A comprehensive literature search was performed using the Medical Subject Heading (MeSH) thesaurus keywords “mesenteric ischaemia”, “bowel ischaemia” and “bowel infarction”. The bibliographies of relevant articles were screened for additional publications. After an initial systematic review of the literature by the whole group, a steering group formulated questions using a modified Delphi process. The evidence was then reviewed to answer these questions, and recommendations formulated and agreed by the whole group.

**Results:**

The resultant recommendations are presented in this paper.

**Conclusions:**

The aim of these guidelines is to provide recommendations for practice that will lead to improved outcomes for patients.

## Introduction

AMI accounts for about 1:1000 acute hospital admissions in Europe and the USA [1]. In Japan, where the incidence of vascular disease is lower, this figure has been estimated at 1:10,000 [2]. The incidence appears to be increasing due to an ageing general population with increasing prevalence of comorbidities. This pre-existing disease worsens the prognosis of intestinal necrosis [3].

The diagnosis of AMI is difficult and it will often go unrecognized as a cause of death. A population-based study from a national general practitioner database in the UK estimated the overall incidence of AMI at 0.63 per 100,000 person years [4], while a population-based study in Sweden with an 87 % autopsy rate estimated the incidence more than twenty times higher at 12.9 per 100,000 person years [5]. Sixty-five percent of acute superior mesenteric artery occlusions were diagnosed at autopsy. The incidence increases exponentially with age and there appears to be an equal incidence in men and women after adjusting for age and gender in the population [5]. While the mean age is around 70 years in most studies, several report cases in their 20 s [2, 6–9].

Four different aetiological forms of AMI have been identified: arterial embolism (EAMI), arterial thrombosis (TAMI), venous thrombosis (VAMI) and non-occlusive mesenteric ischaemia (NOMI). Although they have different clinical and pathophysiological features this does not facilitate early diagnosis of the disease.

Twenty-five percent of the cardiac output goes to the splanchnic circulation at rest increasing to 35 % in the postprandial state. Seventy percent of mesenteric blood flows to the mucosa and submucosa [10] and microscopic changes of ischaemia can be detected within minutes [11]. Although the gut can survive a 75 % reduction in blood flow for up to 12 h without significant injury [12], irreversible bowel damage occurs within 6 h of complete vascular occlusion [13].

Untreated, AMI will cause mesenteric infarction, intestinal necrosis, an overwhelming inflammatory response and death. Early intervention can halt and reverse this process leading to a full recovery while failure to recognize AMI before intestinal necrosis has developed is responsible for the high mortality of the disease [14–16]. Early diagnosis and prompt treatment are the goals of modern therapy, but diagnosis is difficult particularly in the early stages when intervention would be of most benefit. A high index of suspicion in the elderly patient with pain out of proportion to clinical signs and an untreated cardiac arrhythmia is not an adequate baseline. In many studies up to 20 % of patients had no pain recorded [2, 6, 7, 17–24] and in one study 65 % of patients were intubated at the time of referral [25]. Moreover, many patients have peritonitis at presentation.

Although mortality rates have declined over the past 50 years [5, 26] they remain unacceptably high at 50–69 % [6, 27–29]. Overall 26 % of people admitted to hospital with acute mesenteric ischaemia will be alive a year later. However, those patients who are discharged alive have a reasonably good prognosis given the prevalence of significant co-morbidities among that cohort with 84 % alive at one year and 50–77 % at 5 years [18, 30–32]. Ten year survival of almost 30 % has been reported [18, 32]. A median survival of 52 months has been reported in patients with arterial occlusive AMI who survived their acute hospital admission [9].

There are no randomized controlled trials to guide treatment and the published literature contains a high ratio of reviews to original data. Much of that data comes from case reports and often small, retrospective series with no clearly defined treatment criteria.

The aim of these guidelines is to provide recommendations for practice that will lead to improved outcomes for patients.

## Materials and methods

### Definitions

For the purpose of these guidelines, acute mesenteric ischaemia (AMI) is defined as sudden acute arterial or venous occlusion or drop in circulating pressure resulting in insufficient blood flow within the mesenteric circulation to meet the metabolic demands placed upon it. Isolated colonic ischaemia and focal segmental ischaemia secondary to adhesions, hernias or other forms of extrinsic compression are excluded. Chronic mesenteric ischaemia and ischaemic colitis are separate entities and are not considered in these guidelines.

### Search strategy and consensus approach

The European Society for Trauma and Emergency Surgery (ESTES) Study Group was formed in 2013 with the aim of developing guidelines for the management of AMI. Comprehensive computer database literature searches were performed using the indexed online database MEDLINE/PubMed using the Medical Subject Heading (MeSH) thesaurus keywords “mesenteric ischaemia”, “bowel ischaemia” and “bowel infarction”. Searches were limited to human studies. Abstracts from original publications were screened for relevance and full publications evaluated where appropriate. Lists of quoted literature within these articles were also screened for additional relevant publications.

After an initial systematic review of the literature by the whole group, the steering group formulated questions using a modified Delphi process [33]. The evidence was reviewed and recommendations formulated and agreed by the whole group. The classification system used to determine the strength of evidence (LOE) is given in Table 1 [34].

**Table 1:** Classification system used to determine strength of evidence

Level of evidence	Definition
IA	Evidence from meta-analysis of randomized controlled trials
IB	Evidence from at least one randomized controlled trial
IIA	Evidence from at least one controlled study without randomization
IIB	Evidence from at least one other type of quasi-experimental study
III	Evidence from non-experimental descriptive studies such as comparative studies, correlation studies and case controlled-studies
IV	Evidence from expert committee reports or opinions or clinical experience of respected authorities or both

## Presentation and clinical diagnosis

### Which clinical factors should arouse suspicion of AMI in the acute abdomen?

**Answer:** Most patients present with abdominal pain of sudden onset. The early phase of AMI can be characterized by an initial discrepancy between the severity of the abdominal pain and minimal findings on physical examination. Patients can also present with symptoms of nausea, vomiting and initial forced evacuation, early in the course of the disease. The location of pain varies, but as ischemia progresses to infarction, it becomes diffuse. The development of transmural infarction may also be signalled by fever, bloody diarrhoea and shock.

**Background:** The available evidence comes from level II and III studies, with mostly small and retrospective observational case-series. However, they all consider pain as the main symptom in most cases [14, 35]. The classical presentation has been described as pain out of proportion to the findings on clinical examination [10, 19, 36–41] but in 20 to 25 % of patients the initial presentation resembles an acute abdomen from another cause [36, 42, 43]. Other frequent symptoms associated with pain in the early course include nausea (93 %), vomiting (80 %) and diarrhoea (48 %) [19, 40, 41]. Klass’ classical description [44] of the onset of abdominal pain with sudden simultaneous passage of stools as a characteristic sign of AMI is also reported in recent studies [39, 43, 45]. However, these symptoms are not specific for AMI.

Features in the patient’s medical history can be important, particularly in the elderly presenting with 2–3 h of continuous abdominal pain [46]. A retrospective series of 215 AMI patients identified significant comorbidities that predispose to AMI such as ischaemic heart disease, atrial fibrillation, hypertension, diabetes mellitus or renal insufficiency in most patients [47]. Another retrospective series of 47 patients over 13 years observed atrial fibrillation in all 14 patients with arterial embolism and ischemic cardiomyopathy in 18 of 20 cases of arterial thrombosis [48]. As the disease progresses and ischemia leads to intestinal necrosis, pain becomes more diffuse and signs of peritoneal irritation [14] and bloating appear [39]. The patient may develop bloody diarrhoea, fever, signs of shock, multiple organ failure and a reduction in consciousness [37, 39].

**Recommendation:** Acute mesenteric ischaemia (AMI) should be suspected in patients with acute abdominal pain in whom there is no clear diagnosis, particularly when the pain is disproportionate to the physical examination findings and in the elderly with a history of cardiovascular comorbidities (LOE: III).

### Are there any clinical features to distinguish the aetiology of AMI?

**Answer:** Clinical assessment does not reliably distinguish between mesenteric arterial embolism or arterial thrombosis. However, the four aetiological types of AMI have been associated with different characteristics and risk factors (Table 2). EAMI is characterized by a sudden onset of pain and is frequently associated with atrial fibrillation. TAMI has a more indolent course and is often associated with a history of abdominal angina and weight loss suggestive of undiagnosed chronic mesenteric ischaemia. VAMI appears in younger patients, sometimes with several days of mild symptoms. It is associated with hypercoagulable states, cirrhosis, severe pancreatitis, abdominal trauma and advanced malignancy. NOMI is often silent as it occurs in patients that are critically ill and often ventilated.

**Table 2:** Characteristics and risk factors associated with AMI

	Associated comorbidities	Onset of pain	Associated symptoms	Related procedures
EAMI	Heart disease (atrial fibrillation, rheumatic, myocardial infarction, prosthetic valve, ventricular aneurism, Chagas’ disease)	Acute	Diarrhoea, vomiting	Angiography
TAMI	Arteriosclerosis, hypertension, diabetes, hyperlipidemia, dehydration, antiphospholipid syndrome, estrogens	Acute, may be recurrent	Sitophobia, postprandial pain	Vascular surgery (bypass)
VAMI	Hypercoagulable disorders, sickle cell disease, right sided heart failure, DVT, malignancies, hepatitis, pancreatitis, sepsis hepato-splenomegaly, cirrhosis	Gradual	Vague complaints	Recent abdominal surgery
NOMI	Shock, hypovolemia, hypotension, digitalis, diuretics, beta-blockers, alpha-adrenergics, enteral nutrition, critical care support	Either acute or gradual		

**Background:** Embolism is the most frequent cause of mesenteric ischaemia (45 %) [10]. Cardiac ischemia, tachyarrhythmia, rheumatic fever, and other conditions that predispose to the formation of atrial thrombi are risk factors of the disease [35, 36, 38, 42, 49].

Approximately 33 % of patients present with a history of recent embolism [36, 39] and the absence of suitable anticoagulant treatment in these patients should increase suspicion of EAMI [21, 35, 36, 42]. The sudden onset of severe pain with spontaneous emptying of the bowel (vomiting and diarrhoea) but no significant physical findings is a classic sign of an EAMI. If a potential source of emboli can be identified, this “clinical triad” is present in 40–80 % of patients [38].

Arterial thrombosis accounts for approximately 25 % of cases of AMI [10]. These patients usually report prodromal symptoms of mesenteric angina [36] (postprandial abdominal pain, nausea and weight loss) before the acute episode resulting from pre-existent vascular insufficiency [10, 39]. The main risk factors for TAMI are atherosclerotic disease and dyslipidaemia [49, 50]. There may be a history of other vascular events and previous vascular surgery.

Venous thrombosis represents 10 % of cases of patients with acute mesenteric ischaemia and usually occurs in a younger population. While thromboembolic AMI is more common in the over 60 s, VAMI tends to occur in people over 40 [36, 47, 51, 52]. Although occasionally idiopathic, most patients have an identifiable risk factor [10, 51, 53]. Up to 50 % of patients with VAMI report a previous deep venous thrombosis or pulmonary embolism [54]. Other risk factors include hypercoagulability states (protein C and S deficiency, polycythaemia or Leiden factor V mutation), portal hypertension, abdominal trauma, abdominal infection, acute pancreatitis, malignancy, nephrotic syndrome, cirrhosis or splenomegaly [10, 40, 49, 51–53, 55–57]. Leiden factor V mutation is the most common associated hypercoagulability state and is reported in 20–40 % of VAMI cases [40]. Oral contraceptives, pregnancy and the puerperium are risk factors in young women [35, 58]. Venous vascular occlusion is usually peripheral, involving short segments of bowel [53]. The onset of VAMI is characterized by subacute abdominal pain that may develop over a period of up to 2 weeks. Half of the patients complain of nausea and vomiting. Untreated cases may result in portal hypertension with the development of oesophageal varices [39]. VAMI is not usually associated with postprandial syndrome, although bloating, abdominal distension, fever and occult blood in stools may be present [59].

NOMI is responsible for about 20 % of cases of mesenteric ischaemia. It usually occurs in patients that are critically ill, sedated and artificially ventilated and is difficult to recognize. It is poorly understood, but can be explained by a combination of low cardiac output and vasoconstriction. Risk factors for NOMI include age over 50, history of acute myocardial infarction, congestive heart failure, aortic insufficiency, cardiopulmonary bypass, kidney or liver disease or major abdominal surgery. Notably, many patients with NOMI may have none of these factors [10].

The diagnosis should be suspected in patients with mesenteric hypoperfusion secondary to circulatory shock or vasoactive drugs (including amines, cocaine and digitalis) when there is a significant unexpected deterioration in their clinical course [40, 41, 43, 60]. Acute or insidious pain (without defecation), bloating, abdominal distension and the presence of occult blood in the stools are all consistent with NOMI in a critically ill patient [10, 21, 36, 45, 54]. Most patients display signs of sepsis and abdominal distension as a late clinical sign [10].

Mitsuyoshi et al. [61] suggested diagnostic criteria for NOMI in the critically ill patient consisting of 3 of: ileus or abdominal pain, need for catecholamines, episode of hypotension or rising level of transaminases. Although they were able to demonstrate early detection and improved survival, the numbers involved were small.

Large doses of vasopressors alone can cause bowel ischaemia by non-occlusive, low perfusion mechanisms (NOMI). AMI has been associated with drug toxicity. Several case reports link cardiotoxins, such as digitalis, in combination with furosemide-induced fluid depletion, calcium-channel blockers [36], cocaine (linked to arterial thrombosis), organophosphates, ergotamine, phenobarbital, ethylene glycol or tricyclic antidepressants to NOMI in ICU patients. Snake bites have also been associated with NOMI [62, 63].

NOMI has also been described in patients who have undergone the stress of a surgical procedure or trauma and are receiving enteral nutrition in intensive care units. The reported incidence of AMI in these patients is 0.3–8.5 % [10, 64]. The proposed mechanism is an imbalance between demand (created by the enteral feeding) and supply (decreased by systemic hypo-perfusion and mesenteric vasoconstriction).

**Recommendations:** AMI secondary to an arterial embolism (EAMI) should be suspected in patients with atrial fibrillation who have a sudden onset of abdominal pain. AMI resulting from arterial thrombosis (TAMI) should be suspected in patients with evidence of atherosclerotic disease particularly with a recent history of postprandial syndrome. AMI due to venous thrombosis (VAMI) should be suspected in patients with hypercoagulable states. Non-occlusive mesenteric ischaemia (NOMI) should be suspected in critically ill patients with an unexpected deterioration in their clinical condition (LOE: III).

### Can interventional procedures precipitate AMI?

**Answer:** Any procedure which involves vascular manipulation (even unintentional) can precipitate AMI.

**Background:** Acute mesenteric ischaemia has been associated with several procedures and may complicate any abdominal surgery [21]. VAMI is a recognised complication of laparoscopic colorectal surgery. A retrospective analysis over 10 years (1069 colorectal operations) identified 37 (3.5 %) cases of AMI. Inflammatory bowel disease, ulcerative colitis, preoperative therapy with steroids, operative time longer than 220 min, ileoanal pouch anastomosis, total proctocolectomy or postoperative septic complications were associated with thrombosis. The latter two were independent predictors of thrombosis in multivariate analysis. Manipulation of mesenteric vessels and raised intra-abdominal pressure may play a role in the pathophysiology [65].

EAMI may occur when atheromatous plaques are dislodged by angiography of the coronary or cerebral circulation [10]. Aortic catheterization can induce cholesterol embolization [59].

AMI may be seen after colonoscopy. The decreased intravascular volume resulting from fasting and colon preparation, reduction of vascular tone through medications used for conscious sedation and the mechanical effects of colonoscopy may work together to create a low flow state precipitating acute mesenteric ischemia [66]. Possible predisposing conditions include connective tissue disease, advanced age, cardiovascular disease and immunosuppression.

AMI is an infrequent event after coronary bypass (1 %) or valve replacement surgery (0.2–0.4 %). It occurs particularly in older, dehydrated patients who have generalized atherosclerosis, and has a 70–100 % mortality [67]. Several factors (low cardiac output, use of vasopressors and underlying atherosclerotic disease) contribute to severe hypoperfusion and NOMI is the most frequent pathophysiological event [41]. Intra-aortic balloon pumps cause embolic showers particularly if placement involves excessive manipulation of a diseased aorta [50].

**Recommendation:** Unexplained abdominal pain after any invasive procedure, particularly involving vascular manipulation, should lead to suspicion and investigation of AMI (LOE: III).

### Can we predict prognosis at presentation, in order to help the decision making process?

**Answer:** It is difficult to predict prognosis at presentation based exclusively on clinical findings. However, older patients with delayed presentation and abdominal signs of peritonitis or organ failure generally have a worse prognosis. A number of scoring systems have been proposed for AMI, but these have not been validated in large-scale studies.

**Background:** The moribund patient with significant co-morbidities and poor performance status is unlikely to benefit from intervention. A number of factors including admission from a nursing home, or partial dependence, pre-existing ‘do not attempt resuscitation’ order, and class 4 wound before surgery are all associated with increased mortality [68]. So too are coma, artificial ventilation, acute renal failure, chronic obstructive pulmonary disease, myocardial infarction within the preceding 6 months as well as preoperative sepsis, major surgery, emergency procedure, duration of surgery and postoperative complications [68].

Most of the literature considers old age and late diagnosis as bad prognostic factors for arterio-occlusive disease. However, in VAMI longer duration of symptoms before hospital admission may be related to better outcome. With arterio-occlusive AMI a cut off point of 60-65 years and 24 h from the onset of symptoms are associated with better prognosis [7, 14, 21, 30]. One study reported thirty day survival of 81 % for patients with AMI under the age of 71. This fell to 30 % between 71 and 84 and 7 % in the over 84 s [28]. It was associated with increasing rates of non-resectable gangrene in these age groups of 9, 45 and 79 %, respectively.

In a series of patients with AMI mortality was 10.6 % if operated in the first 24 h after onset of symptoms vs. 72.9 % if operated after 24 h [14].

In arterial occlusion, all symptoms but abdominal pain indicate disease progression. The presence of peritoneal irritation is associated with bowel necrosis and worse prognosis [14, 42]. Renal failure and acidosis resulting from shock and sepsis are independent risk factors for mortality [7, 35].

In a retrospective study of 58 patients resection (at first or second look procedure) and no recent major cardiovascular intervention were associated with better survival rates [21]. A larger retrospective series of 124 patients identified older age, bandemia, elevated serum aspartate aminotransferase, increased blood urea nitrogen and metabolic acidosis as independent predictors of death in AMI [7].

APACHE-II and P-POSSUM are not accurate scoring systems for outcomes after emergency surgery and P-POSSUM may under-predict mortality from AMI [69]. However, they may provide a useful indicator of morbidity and mortality. Other scoring systems for predicting outcome from AMI have shown early promise, but require validation in further studies [2, 30, 68].

**Recommendation:** Management decisions should not be based exclusively on clinical findings. However, patients with advanced age, late presentation, peritonitis and signs of organ failure are unlikely to benefit from invasive procedures and should be considered for palliative care only (LOE: III).

## Diagnosis

### Are there sensitive and specific laboratory tests that can be used for early detection of AMI?

**Answer:** There is no specific laboratory test that can be routinely used for early detection of AMI.

**Background:** The ideal plasma biomarker for AMI should be specific for bowel ischemia and highly sensitive. As ischemia starts from the mucosa and progresses to the serosa, a mucosa-derived marker would be most useful for early diagnosis [70]. The most promising plasma markers are intestinal fatty acid binding protein (I-FABP) and α-glutathione S-transferase (GST), originating from mucosa of the small bowel and D-lactate which is produced by gut bacterial organisms such as *Escherichia coli* [71, 72] These markers may have a potential use as early diagnostic tools in AMI, but none of them has yet entered routine clinical practice.

Serum L-lactate is a specific marker of tissue hypo-perfusion [73]. The liver can clear large quantities of L-lactate from the porto-mesenteric circulation and as a result serum lactate level does not correlate with intestinal infarction [74]. Lactic acidosis develops late in the course of AMI with extensive transmural infarction and tissue hypoperfusion due to sepsis. At that point mortality is already in the order of 75 % [75].

The fibrinolytic marker D-dimer does not differentiate patients with AMI from those with non-acute mesenteric ischemia and that there is no difference in D-dimer levels between patients with resectable and irresectable bowel necrosis [76].

The most common laboratory abnormalities found in AMI are hemoconcentration, leukocytosis, metabolic acidosis with high anion gap and lactate concentration, and high levels of serum amylase, aspartate aminotransferase, lactate dehydrogenase and creatine phosphokinase. None of these is sufficiently sensitive or specific to diagnose AMI [10, 77].

**Recommendations:** A normal serum lactate level does not exclude AMI and should not be used for diagnosis (LOE: III).

Routine laboratory tests reflect disease progression in AMI, but should not be used for diagnostic purposes (LOE: III).

### What is the most sensitive and specific test for the detection of AMI?

**Answer:** The most sensitive and specific diagnostic tool is biphasic multidetector computed tomography (MDCT) with intravenous contrast.

**Background:** The diagnostic work-up of AMI requires a non-invasive test that confirms the diagnosis and differentiates it from other abdominal pathology.

A plain abdominal radiograph has no role in the early detection of AMI, since a normal radiograph does not exclude the diagnosis. Radiographic signs in patients with AMI are nonspecific and appear when bowel infarction has already occurred [78].

Percutaneous angiography has been replaced as the gold standard for diagnosis of suspected AMI by MDCT. No relevant trials supporting the use of angiography for diagnosis can be found in the recent literature [79, 80]. MDCT should be the first line imaging method in suspected AMI due to its high diagnostic accuracy [81–85]. This imaging modality also allows other causes of acute abdominal pain to be excluded.

In this context biphasic CT involves the acquisition of scans in the arterial and venous phases. Scans are also acquired before intravenous contrast is given. Pre-contrast scans detect vascular calcification, hyper-attenuating intravascular thrombus and intramural haemorrhage, while contrast-enhanced CT allows the identification of thrombus in the mesenteric arteries and veins, abnormal enhancement of the bowel wall and the presence of embolism or infarction of other organs. Sagittal reconstructions are used to assess the origin of the mesenteric arteries [83].

The use of oral contrast is generally not feasible in patients with AMI. The transit time for oral contrast through the bowel will delay definitive treatment in AMI and the associated vomiting and an adynamic ileus limit the useful passage of oral contrast material [83, 86].

MDCT has a high specificity and sensitivity [82]. A meta-analysis of six primary studies using MDCT on 619 cases with suspected AMI showed a pooled sensitivity of 93.3 % (95 % confidence interval 82.8, 97.6 %) and a pooled specificity of 95.9 % (95 % confidence interval 91.2, 98.2 %). Among confirmed cases of AMI, final clinical diagnoses were EAMI/TAMI in 69 % of patients, VAMI in 15 % and NOMI in 16 % [87].

The high diagnostic accuracy of MDCT is based on specific features of AMI such as occlusion of mesenteric vessels and the non-specific appearance of bowel wall thickening and intestinal pneumatosis [88]. Some of these features, like diameter of the small mesenteric vessels or gas bubbles in intestinal pneumatosis, are no larger than 1–2 mm which requires a high spatial resolution achieved with MDCT with a collimation of 1.25–2.5 mm in the arterial or the portal venous phase [83, 88]. Pneumatosis Intestinalis (presence of air within the bowel wall) usually indicates transmural infarction in AMI, particularly if it is associated with portomesenteric venous gas, but this sign is not specific for either infarction or ischemia.

NOMI represents the most difficult diagnostic challenge in AMI; usually these critically ill patients are sedated and intubated and diagnosis may rely only on clinical suspicion and imaging studies. The administration of intravenous contrast may be contraindicated, hence a lesser sensitivity and utility of MDCT [89]. The diagnosis of NOMI should be confirmed by selective catheter angiography [90, 91]. On CT, the bowel wall of the involved segments may be normal or thickened and the pattern of enhancement is variable ranging from absent or diminished to increased, while fat stranding of the mesentery and ascites are usually visible [83].

The CT findings in NOMI are poorly understood [86] although in two published papers, with a total of 6 cases, that specifically addressed the CT findings of surgically and pathologically proven NOMI [92, 93] the reported CT findings consisted of diffuse narrowing of the SMA, poor visualization of its secondary branches, bowel distension, thin and poorly enhancing bowel wall, and intestinal pneumatosis which are similar to the findings of acute occlusive AMI, except for the absence of thrombus/embolus. The most common features associated with AMI are described in Table 3.

**Table 3:** Radiological features associated with AMI

Characteristic	EAMI–TAMI	VAMI	NOMI
Bowel wall	Thinning (“paper thin wall”), no change, or thickening with reperfusion	Thickening	No change or thickening with reperfusion
Attenuation of bowel wall on unenhanced CT	Not characteristic	Low with oedema; high with haemorrhage	Not characteristic
Enhancement of bowel wall on contrast-enhanced CT	Diminished, absent, target appearance or high with reperfusion	Diminished, absent, target appearance, or increased	Diminished, absent, heterogeneous in distribution
Bowel dilatation	Not apparent	Moderate to prominent	Not apparent
Mesenteric vessels	Defect or defects in arteries, arterial occlusion, SMA >SMV in diameter	Defect or defects in veins, venous engorgement	No defect, arterial constriction
Mesentery	Not hazy until mesenteric infarction occurs	Hazy with ascites	Not hazy until mesenteric infarction occurs

Modified from Furukawa et al. [83]

*SMA* superior mesenteric artery, *SMV* superior mesenteric vein

**Recommendations:** In cases of suspected AMI, multidetector computerised tomography scanning (MDCT) with intravenous contrast should be performed immediately. The use of oral contrast will add significant delay to the MDCT and should be avoided (LOE: III).

Percutaneous angiography should not be used for initial diagnosis of AMI except where NOMI is suspected (LOE: III).

### Is there a role for diagnostic laparoscopy in patients with AMI?

**Answer:** Laparoscopy can be used for diagnosis and second-look.

**Background**: Several articles with small series of patients address the use of laparoscopy in AMI [94–96]. In cases of uncertainty regarding the diagnosis or the extent of bowel necrosis, laparoscopy can offer a minimally invasive way to asses it, even in the ICU (bedside laparoscopy). Laparoscopic second-look procedures can be offered in a planned or on-demand fashion. However, there is no strong evidence to support the routine use of laparoscopy in AMI.

**Recommendations:** There is insufficient evidence to support the routine use of laparoscopy in AMI (LOE: IV).

## Treatment

### What are the goals of resuscitation and which fluid should be used?

**Answer:** The main goal of resuscitation is the restoration of adequate tissue/organ perfusion. Supplementary oxygen should be given and crystalloids are the fluid of choice.

**Background:** The principal goals of managing the patient with AMI can be summarized with the 3 “Rs”: Resuscitation, Rapid diagnosis and early Revascularization before there is significant progression of the systemic inflammatory response. The main goal of fluid resuscitation is the restoration of adequate tissue/organ perfusion [97–99]. Supplementary oxygen should be given [100] and the evaluation of organ delivery of oxygen assessed using accepted clinical markers such as peripheral perfusion, mental status, and urine output [97, 100].

A recent Cochrane Review [101] has shown no advantage of colloids over crystalloids in reducing mortality when used for intravenous fluid resuscitation. The choice of fluid will have a cost implication, and colloids are more expensive than crystalloids. As there is no evidence that colloids improve survival rates or reduce morbidity, crystalloids should be preferred on economic grounds. Evidence suggests that the use of hydroxyethyl starch might increase mortality and should be avoided until further proof of safety can be provided [101–105].

**Recommendations**: Supplementary oxygen should be given immediately (LOE: III).

Fluid volume status should be quickly assessed and fluid replacement should start promptly but should not delay diagnosis and intervention (LOE: IV).

Crystalloids should be used for fluid replacement, hydroxyethyl starch should be avoided (LOE: Ia).

### Is there a role for vasopressor drugs?

**Answer:** Vasopressors reduce splanchnic perfusion and should be avoided in AMI whenever possible. Pressors also reduce the effectiveness of angiography. However, when required consideration should be given to using a pressor which has less effect on mesenteric blood flow.

**Background:** AMI results from insufficient blood flow within the mesenteric circulation to meet the metabolic demands placed upon it. This may be confounded by hypovolaemia and circulatory failure. The use of vasopressors to improve cardiac function should be balanced against the adverse effect of further splanchnic vasoconstriction [3, 106]. Consideration should be given to the use of drugs such as dobutamine, low dose dopamine and milrinone, which have been shown to have less impact on mesenteric blood flow [39]. Vasopressors should not be used until the patient has been adequately volume resuscitated. Cardiac output can also be improved by rate control in atrial fibrillation. However, digoxin and other cardiac glycosides also reduce flow in the splanchnic circulation and should be avoided for the control of atrial fibrillation/flutter in patients with AMI [40, 96, 98].

**Recommendations:** Vasopressor drugs should be avoided in AMI. If vasopressor drugs are required after adequate volume replacement, preference should be given to those with minimal effect on the splanchnic circulation (LOE: IV).

Cardiac glycosides should not be used as first line treatment of atrial fibrillation/flutter in AMI (LOE: IV).

### Is there a role for antibiotics?

**Answer:** AMI affects the mucosa first and bacterial translocation is an early event in the progression of AMI. Broad-spectrum antibiotic cover should be given.

**Background:** Loss of the integrity of the mucosal barrier facilitates bacterial translocation and occurs in the early stages of AMI. Although no specific studies have examined the role of prophylactic antibiotics in AMI, broad spectrum antibiotics (such as a penicillin or a third generation cephalosporin in combination with metronidazole) would be expected to reduce the consequences of bacterial translocation and should be given early [107].

**Recommendation:** Broad spectrum antibiotics should be administered early in the course of AMI (LOE: IV).

### What is the specific treatment for AMI?

#### Arterial embolism (EAMI)

**Answer:** Open embolectomy is widely used in this scenario. However, if expertise and appropriate resources are available, and there is no evidence of bowel necrosis, endovascular techniques should be attempted.

**Background:** The established treatment is open surgical embolectomy. However, an increasing number of cases of successful percutaneous treatment have been reported with overall results comparable to the open approach [91, 106].

Open embolectomy is usually performed via a midline incision approaching the SMA just below the pancreas at the mesenteric root [98]. A transverse arteriotomy is then made after proximal and distal clamping and embolectomy catheters are used to clear the artery proximally and distally. High pressure proximal flushing should be avoided so as not to dislodge the thrombus in the aorta and produce further emboli. After completing the thrombectomy the artery should be flushed gently with heparinized saline. The arteriotomy is then closed in a standard fashion.

Endovascular embolectomy is achieved by percutaneous mechanical aspiration [108] or thrombolysis [109, 110] and permits percutaneous transluminal angioplasty (PTA), if necessary, with or without stenting [111, 112].

**Recommendation:** In cases where immediate surgical intervention is not required the decision to perform endovascular or open vascular surgery for EAMI should be determined by the personal experience and technical capabilities of the surgeon and the available resources (LOE: IV).

When EAMI is identified during a laparotomy an open embolectomy should be performed (LOE: IV).

#### Arterial thrombosis (TAMI)

**Answer:** Endovascular treatment should be the first choice for TAMI whenever possible.

**Background:** Endovascular treatment should be considered as soon as possible for acute thrombosis of a diseased superior mesenteric artery (SMA) [91, 98, 106, 113, 114]. This should be performed before intestinal infarction occurs and when the ischaemia is potentially reversible. The commonest interventions are PTA and stenting. Other endovascular techniques include percutaneous aspiration thrombectomy, local fibrinolysis or intra-arterial drug perfusion (such as heparin or papaverine).

If surgery is needed to resect ischaemic bowel before vascular intervention, or when percutaneous treatment has failed, retrograde open mesenteric stenting (ROMS) has been suggested [113].

Conventional bypass surgery is another option [98]. There are a variety of bypass procedures, providing either antegrade or retrograde flow, with vein or synthetic grafts. An antegrade bypass from supraceliac aorta to superior mesenteric trunk using an endogenous vein graft will give the best result. An alternative approach would be a renal-mesenteric bypass. However, the most practical option for proximal mesenteric atherosclerotic occlusive disease is a retrograde bypass from common iliac with a synthetic graft.

Where vascular expertise is not available it may be reasonable to resect the necrotic bowel first and transfer the patient for urgent interventional angiography or vascular surgery [91].

**Recommendations:** When bowel integrity has not been compromised, endovascular techniques should be performed as first line treatment for TAMI (LOE: III).

When a laparotomy has been performed for TAMI the choice of vascular intervention will depend on available resources and expertise (LOE: IV).

When vascular expertise is not available it may be reasonable to resect the necrotic bowel first and transfer the patient for urgent interventional angiography or vascular surgery (LOE: III).

#### Venous ischaemia (VAMI)

**Answer:** The first line treatment for mesenteric venous thrombosis is anticoagulation.

**Background:** VAMI is usually managed without surgery. Anticoagulation with a continuous infusion of unfractionated or low molecular weight heparin is the first line treatment for VAMI and should also be started in patients diagnosed at operation [80, 91, 98, 106, 115–117]. Isolated thrombosis of the superior mesenteric vein is usually compensated by sufficient collateral circulation. However, additional complete thrombosis of the portal vein leads to venous infarction of segments of small intestine of varying severity and may require laparotomy.

Patients who deteriorate during medical treatment can be considered for endovascular treatment. Many techniques have been described such as transjugular intrahepatic portosystemic shunting (TIPS) with mechanical aspiration thrombectomy and direct thrombolysis, percutaneous transhepatic thrombolysis, indirect thrombolysis via the SMA and thrombolysis via a surgically placed superior mesenteric vein (SMV) catheter.

A number of case-series advocate prompt direct thrombolysis on the basis of reported reduction in both early complications (infarction) and late sequelae (portal hypertension) [116]. Contraindications to thrombolytic treatment are well established and can be divided into absolute (central nervous system tumours, recent haemorrhagic stroke, gastrointestinal (GI) bleed and uncontrolled hypertension) and relative (pregnancy, remote history of GI bleeding, and recent major surgery).

**Recommendations:** Systemic anticoagulation should be started as soon as possible in VAMI (LOE: III).

Endovascular intervention should be offered to patients with VAMI who deteriorate during medical therapy (LOE: IV).

#### Non-occlusive mesenteric ischaemia (NOMI)

**Answer:** The first line treatment for NOMI is medical therapy with direct infusion of vasodilators into the SMA.

**Background:** The treatment of NOMI is based on correcting the clinical or pharmacological conditions that generate splanchnic vasoconstriction, improving mesenteric perfusion and early recognition and resection of infarcted bowel [99].

The main factor in improving mesenteric perfusion is the restoration of circulatory volume and hemodynamic stability coupled with the use of vasodilators administered directly into the SMA in order to reverse the intestinal vasospasm that causes ischemia.

The diagnosis of NOMI should be confirmed by selective mesenteric angiography and, if there are no contraindications, the infusion of vasodilators directly into the SMA [90]. The best vasodilator drug appears to be prostaglandin E1 (alprostadil) given as a 20 mcg bolus followed by 60–80 mcg/24 h infusion [90]; papaverine (30–60 mg/h) has been shown to reduce the mortality rate for NOMI from 70 to 50–55 % and is an acceptable alternative [1, 10]. Other prostacyclin analogues have also been used [61].

The decision to intervene surgically is based on the presence of peritonitis, perforation, or overall worsening of the patient’s condition [99].

**Recommendation:** NOMI should be managed by correcting the underlying cause wherever possible and improving mesenteric perfusion by direct infusion of vasodilators. Infarcted bowel should be excised (LOE: III).

### How should a patient with peritonitis secondary to AMI be managed?

**Answer:** The common feature in the management of AMI is the need for surgical exploration in the presence of peritonitis. Patients with AMI and signs of peritonitis should undergo immediate surgery if comorbidities and clinical condition make curative treatment possible. Surgical intervention should also be considered if the patient’s condition deteriorates. With severe pre-existing medical conditions, pre-terminal state or extreme old age, the decision to proceed to laparotomy may not be appropriate.

**Background:** Across the literature there is agreement that peritonitis secondary to bowel necrosis mandates surgery without delay if curative treatment is to be achieved [98, 106]. All other priorities (type of AMI, level of occlusion, etc.) are secondary and should be managed accordingly.

**Recommendation:** Patients with AMI and signs of peritonitis should undergo immediate surgery if comorbidities and clinical condition make curative treatment possible (LOE: III).

Patients considered unsalvageable should have palliative care (LOE: IV).

### What is the role of Damage Control Surgery in AMI?

**Answer:** Damage Control (DCS) is the surgical modality of choice in the critically ill patient with AMI.

**Background:** Patients with AMI who have severe sepsis or septic shock and undergo life-saving surgery should have a damage control approach. [118–121]. This includes immediate laparotomy with resection of ischaemic bowel (and no anastomosis or stoma), open thrombectomy (if indicated), transfer to the intensive care unit to continue resuscitation prior to planned definitive procedures [122]. A temporary abdominal closure via a negative pressure wound therapy (NPWT) device should be considered since they have been shown to promote wound healing and facilitate subsequent abdominal closure [123, 124]. A scheduled ‘second look’ procedure should be performed within 48 h [125, 126].

**Recommendation:** Patients with AMI who have severe sepsis or septic shock should undergo life-saving damage control surgery (DCS) (LOE: III).

### How should bowel viability be assessed at operation?

**Answer:** Assessment is based on the macroscopic appearance of the bowel: its colour, motility and bleeding of cut ends.

**Background:** Necrotic bowel will be clearly identifiable at laparotomy [122, 127]. The viability of the remaining bowel should be determined after the patient has been adequately resuscitated and any resection/revascularization performed [122]. In the acute setting, hypotension, vascular impairment and the concurrent use of vasopressors may confound this assessment and could result in excessive resection [106]. It is often better to adopt a damage limitation approach and schedule a second-look procedure under these circumstances.

Intra-operative assessment with Doppler ultrasound of the vascular arcade, [128, 129], fluorescein angiography [127, 130] and indocyanine green angiography [131] have shown promise, but not been used extensively.

**Recommendations:** Necrotic bowel should be removed if the patient is considered salvageable (LOE: III).

Ischemic bowel should be reassessed after adequate fluid resuscitation and revascularization. If any doubt remains about the viability of the bowel, a second-look procedure should be performed (LOE: IV).

### What limits should be observed in extensive bowel resection?

**Answer:** The removal of large amounts of small bowel can result in short bowel syndrome (SBS) and intestinal failure. SBS is associated with poor quality of life and a morbidity/mortality which increases with age and comorbidities. Thus extensive resections should be carefully considered.

**Background:** Vascular occlusion in TAMI usually occurs in the proximal SMA and the consequent ischemia may extend from the terminal portion of the duodenum to the transverse colon [36]. In contrast, in EAMI most emboli lodge in the superior mesenteric artery distal to the origin of the middle colic artery. This maintains perfusion of the inferior pancreaticoduodenal arteries and spares the proximal jejunum from ischaemia [10, 36, 132]. However, up to 15 % of emboli in EAMI lodge at the origin of the SMA causing more extensive ischaemia.

Short bowel syndrome requiring total parenteral nutrition (TPN) has been reported in 13–31 % of long-term survivors of AMI [9, 21, 31] and weight loss has been documented in 38 % [31]. SBS generally occurs when less than 200 cm of functioning bowel remains [106].

Restoring bowel continuity will improve the functional results of extensive small bowel resection and may avoid long term TPN. It has been suggested that while permanent intestinal failure is likely with a residual 100 cm of small bowel and an end jejunostomy, the need for permanent TPN may be avoided with a minimum 65 cm of jejunum and a jejunocolic anastomosis, and 35 cm where the ileocaecal region is preserved and a jejunoileal anastomosis performed [133].

When a residual small bowel length of 200 cm cannot be obtained, careful consideration should be given to the significant risk of resection, particularly in the elderly patients and in those with significant co-morbidities. In younger patients the option of long term parenteral nutrition or the option of subsequent intestinal transplantation could make a more extensive resection reasonable.

**Recommendation:** The benefits of extensive small bowel resection should be balanced against the resultant quality of life, morbidity and mortality especially in elderly patients. Restoration of bowel continuity following extensive resection will improve functional results and may avoid the need for long term TPN (LOE: III).

### When is the most appropriate time to perform an anastomosis in a patient with AMI who needs bowel resection?

**Answer:** An anastomosis should be performed only in adequately resuscitated and stable patients when there is no question about the viability of the bowel.

**Background:** Bowel anastomosis should be avoided in the presence of severe sepsis or septic shock or when the patient is inadequately resuscitated. Where limited necrosis is found, there is no doubt about the viability of the remaining bowel, the patient has been adequately resuscitated and there is no evidence of shock, some authors advocate anastomosis with a planned second-look [94]. Laparoscopy can be useful in this circumstance [126]. There are no published studies that compare anastomotic techniques in AMI.

Stomas avoid the risks of anastomotic failure and permit easy examination of the bowel by inspection and/or endoscopy [106]. A mucous fistula of the distal bowel may be used for re-feeding in patients with very proximal jejunostomies [134]. Continuity can be restored later if appropriate.

**Recommendation:** Anastomosis should be avoided in patients with shock or multiple organ dysfunction (LOE: III).

If during a second look procedure there is still doubt about the viability of the bowel it should be exteriorised (LOE: IV).

### What is the role of second-look laparotomy?

**Answer:** The second-look laparotomy plays an important role in AMI patients treated with curative intent and is the natural consequence of DCS.

**Background:** The use of second look procedures is associated with a significant reduction in morbidity (p = 0.002) [9] and mortality in selected patients leading to resection of ischaemic bowel in 38–71 % of cases [14, 17, 18, 24, 29, 126, 135]. It is currently the best method to assess bowel viability after revascularization and resuscitation and may be the best time to perform an anastomosis and to close the abdomen after DCS.

**Recommendations:** After DCS a planned second-look procedure should be performed within 48 h (LOE: III).

A scheduled second-look should also be considered when there is concern about the possible progression of bowel ischemia and there should be a low threshold for an “on demand” second look procedure in AMI particularly when a bowel anastomosis has been performed (LOE: III).

### Can we improve outcomes in terms of mortality and morbidity?

**Answer:** Outcomes for AMI can be improved by rapid diagnosis, optimisation and intervention by revascularization with or without resection.

**Background:** Mortality increases dramatically when symptoms have been present for more than 24 h in AMI. Numerous studies have shown that mortality is lowest if intervention is performed within 12 h of the onset of symptoms. [18, 22, 25, 58, 136–139]. This is supported by experimental work which has shown gut viability to be 100 % within the first 12 h of ischaemia. This drops to 54 % between 12 and 24 h and 18 % beyond 24 h [140].

The development of symptoms in VAMI is usually slower than the other forms of AMI. A small retrospective study of VAMI over 23 years suggested that patients presenting within 3 days of the onset of symptoms had poorer outcomes than those presenting with symptoms of longer duration. The first group were more likely to undergo laparotomy within 12 h of hospital admission (83 vs. 20 %) [52]. This reflected rapid disease progression in the group that presented sooner. In studies comparing the four aetiological types of AMI, VAMI has the lowest mortality (11–30 %) [5, 22, 30].

**Recommendation:** To improve outcomes in AMI a prompt diagnosis should be achieved and revascularization performed within 12 h from the onset of symptoms. Resection of non-viable bowel should be performed without delay (LOE: III).

### Is there a role for prevention?

**Answer:** The diagnosis of AMI is difficult and often delayed or overlooked. Primary prevention should be targeted at managing the risk factors that predispose it. There is little direct evidence for the benefit of secondary prophylaxis of AMI.

**Background:** Since TAMI is usually a marker for advanced systemic atherosclerosis, it would seem reasonable, where appropriate, to advise these patients to stop smoking, modify their diet, lose weight and exercise adequately. Diabetes and hypertension should be treated and the use of statins and antiplatelet therapy considered [141].

Up to 60 % of patients with TAMI have previous symptoms of chronic mesenteric ischaemia (CMI) [9]. The mortality of elective surgical intervention for CMI in selected patients has been shown to be significantly lower than the same procedures performed for TAMI [32]. However, there is little evidence that elective intervention for CMI prevents TAMI.

One-third of patients with EAMI have inadequately treated atrial fibrillation at presentation [9] and a similar number will have further emboli [3]. These patients should be considered for anticoagulation [31].

Cho et al. advocate ‘vigilant surveillance and preservation of SMA patency’ after vascular reconstruction [32]. Although numbers were small, 60 % (3 of 5) of patients who were treated for graft stenosis with stent/angioplasty or further reconstruction had a successful outcome. However, all of these occlusions were symptomatic.

VAMI is usually secondary to a hypercoagulable state [3]. These patients should be investigated for thrombophilia and treated accordingly.

**Recommendations:** All patients with AMI should be advised to make appropriate lifestyle changes to reduce the consequences of vascular disease. Diabetes and hypertension should be treated (LOE: IV).

Patients with proven CMI should be considered for elective revascularization (LOE: IV).

Unless contraindicated, patients with emboli should be treated with life-long anticoagulation to reduce the risk of recurrence (LOE: IV).

Patients with mesenteric artery thrombosis are at high risk of coronary thrombosis and they should be offered anticoagulants or antiplatelet therapy and a statin to reduce this risk (LOE: IV).

Patients with mesenteric venous thrombosis should be investigated for thrombophilia and treated accordingly. Where indicated, they should have a minimum of 6 months of anticoagulation (LOE: III).

Patients who have vascular stents or bypass procedures should have graft surveillance or stent follow up with duplex scanning or CT angiography to enable early detection and management of stenosis and occlusion (LOE: IV).

## Summary of recommendations

1. Acute mesenteric ischaemia (AMI) should be suspected in patients with acute abdominal pain in whom there is no clear diagnosis, particularly when the pain is disproportionate to the physical examination findings and in the elderly with a history of cardiovascular comorbidities (Level of evidence: III).
2. AMI secondary to an arterial embolism (EAMI) should be suspected in patients with atrial fibrillation who have a sudden onset of abdominal pain. AMI resulting from arterial thrombosis (TAMI) should be suspected in patients with evidence of atherosclerotic disease particularly with a recent history of postprandial syndrome. AMI due to venous thrombosis (VAMI) should be suspected in patients with hypercoagulable states. Non-occlusive mesenteric ischaemia (NOMI) should be suspected in critically ill patients with an unexpected deterioration in their clinical condition (Level of evidence: III).
3. Unexplained abdominal pain after any invasive procedure, particularly involving vascular manipulation, should lead to suspicion and investigation of AMI (Level of evidence: III).
4. Management decisions should not be based exclusively on clinical findings. However, patients with advanced age, late presentation, peritonitis and signs of organ failure are unlikely to benefit from invasive procedures and should be considered for palliative care only (Level of evidence: III).
5. A normal serum lactate level does not exclude AMI and should not be used for diagnosis (Level of evidence: III).
6. Routine laboratory tests reflect disease progression in AMI, but should not be used for diagnostic purposes (Level of evidence: III).
7. In cases of suspected AMI, multidetector computerised tomography scanning (MDCT) with intravenous contrast should be performed immediately. The use of oral contrast will add significant delay to the MDCT and should be avoided (Level of evidence: III).
8. Percutaneous angiography should not be used for initial diagnosis of AMI except where NOMI is suspected (Level of evidence: III).
9. There is insufficient evidence to support the routine use of laparoscopy in AMI (Level of evidence: IV).
10. Supplementary oxygen should be given immediately to all patients with AMI (Level of evidence: III).
11. Fluid volume status should be quickly assessed and fluid replacement should start promptly but should not delay diagnosis and intervention (Level of evidence: IV).
12. Crystalloids should be used for fluid replacement, hydroxyethyl starch should be avoided (Level of evidence: Ia).
13. Vasopressor drugs should be avoided in AMI. If vasopressor drugs are required after adequate volume replacement, preference should be given to those with minimal effect on the splanchnic circulation (Level of evidence: IV).
14. Cardiac glycosides should not be used as first line treatment of atrial fibrillation/flutter in AMI (Level of evidence: IV).
15. Broad spectrum antibiotics should be administered early in the course of AMI (Level of evidence: IV).
16. In cases where immediate surgical intervention is not required the decision to perform endovascular or open vascular surgery for EAMI should be determined by the personal experience and technical capabilities of the surgeon and the available resources (Level of evidence: IV).
17. When EAMI is identified during a laparotomy an open embolectomy should be performed (Level of evidence: IV).
18. When bowel integrity has not been compromised, endovascular techniques should be performed as first line treatment for TAMI (Level of evidence: III).
19. When a laparotomy has been performed for TAMI the choice of vascular intervention will depend on available resources and expertise (Level of evidence: IV).
20. When vascular expertise is not available it may be reasonable to resect the necrotic bowel first and transfer the patient for urgent interventional angiography or vascular surgery (Level of evidence: III).
21. Systemic anticoagulation should be started as soon as possible in VAMI (Level of evidence: III).
22. Endovascular intervention should be offered to patients with VAMI who deteriorate during medical therapy (Level of evidence: IV).
23. NOMI should be managed by correcting the underlying cause wherever possible and improving mesenteric perfusion by direct infusion of vasodilators. Infarcted bowel should be excised (Level of evidence: III).
24. Patients with AMI and signs of peritonitis should undergo immediate surgery if comorbidities and clinical condition make curative treatment possible (Level of evidence: III).
25. Patients considered unsalvageable should have palliative care (Level of evidence: IV).
26. Patients with AMI who have severe sepsis or septic shock should undergo life-saving damage control surgery (DCS) (Level of evidence: III).
27. Necrotic bowel should be removed if the patient is considered salvageable (Level of evidence: III).
28. Ischemic bowel should be reassessed after adequate fluid resuscitation and revascularization. If any doubt remains about the viability of the bowel, a second-look procedure should be performed (Level of evidence: IV).
29. The benefits of extensive small bowel resection should be balanced against the resultant quality of life, morbidity and mortality especially in elderly patients. Restoration of bowel continuity following extensive resection will improve functional results and may avoid the need for long term TPN (Level of evidence: III).
30. Anastomosis should be avoided in patients with shock or multiple organ dysfunction (Level of evidence: III).
31. If during a second look procedure there is still doubt about the viability of the bowel it should be exteriorised (Level of evidence: IV).
32. After DCS a planned second-look procedure should be performed within 48 h (Level of evidence: III).
33. A scheduled second-look should also be considered when there is concern about the possible progression of bowel ischemia and there should be a low threshold for an “on demand” second look procedure in AMI particularly when a bowel anastomosis has been performed (Level of evidence: III).
34. To improve outcomes in AMI a prompt diagnosis should be achieved and revascularization performed within 12 h from the onset of symptoms. Resection of non-viable bowel should be performed without delay (Level of evidence: III).
35. All patients with AMI should be advised to make appropriate lifestyle changes to reduce the consequences of vascular disease. Diabetes and hypertension should be treated (Level of evidence: IV).
36. Patients with proven CMI should be considered for elective revascularization (Level of evidence: IV).
37. Unless contraindicated, patients with emboli should be treated with life-long anticoagulation to reduce the risk of recurrence (Level of evidence: IV).
38. Patients with mesenteric artery thrombosis are at high risk of coronary thrombosis and they should be offered anticoagulants or antiplatelet therapy and a statin to reduce this risk (Level of evidence: IV).
39. Patients with mesenteric venous thrombosis should be investigated for thrombophilia and treated accordingly. Where indicated, they should have a minimum of 6 months of anticoagulation (Level of evidence: III).
40. Patients who have vascular stents or bypass procedures should have graft surveillance or stent follow up with duplex scanning or CT angiography to enable early detection and management of stenosis and occlusion (Level of evidence: IV).
